# Undercoverage of lateral trochlear resection is correlated with the tibiofemoral alignment parameters in kinematically aligned TKA: a retrospective clinical study

**DOI:** 10.1186/s12891-021-04064-4

**Published:** 2021-02-17

**Authors:** Zhiwei Wang, Liang Wen, Liang Zhang, Desi Ma, Xiang Dong, Tiebing Qu

**Affiliations:** 1 Department of Orthopedic Surgery, Beijing Chaoyang Hospital, Capital Medical University, 100020 Beijing, China; 2 Beijing Naton Medical Technology Innovation Center Co., Ltd, 100038 Beijing, China; 3 The Center of Diagnosis and Treatment for Joint Disease, Rehabilitation Research Center, 100068 Beijing, China

**Keywords:** Total knee arthroplasty, Osteoarthritis, Kinematic alignment, Trochlea, Anatomy

## Abstract

**Background:**

A mismatch between the femoral component and trochlear resection surface is observed in kinematically aligned total knee arthroplasty (KA-TKA) when conventional prostheses are employed. This mismatch is mainly manifested in the undercoverage of the lateral trochlear resection surface. The aim of the present study was to assess the relationship between the mismatch and the alignment parameters of the tibiofemoral joint.

**Methods:**

Forty-five patients (52 knees) who underwent KA-TKA in our hospital were included. Patient-specific instrumentation was used in 16 patients (16 knees), and conventional instruments with calipers and other special tools were employed in the other 29 patients (36 knees). The widths of the exposed resection bone surface at the middle (MIDexposure) and distal (DISexposure) levels on the lateral trochlea were measured as dependent variables, whereas the hip-knee-ankle angle (HKAA), mechanical lateral distal femoral angle (mLDFA), joint line convergence angle (JLCA), medial proximal tibial angle (MPTA) and transepicondylar axis angle (TEAA) were measured as independent variables. Correlation analysis and subsequent linear regression were conducted among the dependent variables and various alignment parameters of the tibiofemoral joint.

**Results:**

The incidence of undercoverage of the lateral trochlear resection surface was 86.5 % with MIDexposure and DISexposure values of 2.3 (0–6 mm) and 2.0 (0–5 mm), respectively. The widths of the two levels of exposed bone resection were significantly correlated with mLDFA and HKAA but were not related to TEAA.

**Conclusions:**

The undercoverage of the trochlear resection surface in KA-TKA is mainly correlated with the degree of valgus of the distal femoral joint line. The current study suggests that this correlation should be considered in the development of KA-specific prostheses.

## Background

Total knee arthroplasty (TKA) can significantly alleviate the pain of patients with end-stage knee osteoarthritis (OA) and improve knee joint function and quality of life [1, 2]. TKA has achieved tremendous success in the past few decades. In terms of alignment options, mechanical alignment (MA), as a standard surgical technique, has won the approval of the majority of surgeons and technical support from almost all knee prosthesis manufacturers. The aim of MA is to restore the “neutral” alignment of the lower extremities; however, the alignment philosophy of MA conflicts with the “natural” alignment of the normal population [3]. Therefore, some studies supposed that the surgical technical requirements of MA are related to the high dissatisfaction rate of patients after TKA [4–6]. Kinematical alignment (KA) is dedicated to restoring the anatomical geometry of the tibiofemoral joint and the laxity of the joint to that which it would have been before the onset of arthritis [7] and abandoning the “neutral” alignment of the MA. KA has attracted widespread attention in recent years [8–10].

Although kinematically aligned TKA (KA-TKA) strives to restore the morphology of the natural tibiofemoral joint, the use of conventional prostheses to perform KA-TKA cannot take the patellofemoral joint into full consideration [11]. Therefore, whether KA-TKA can cause unpredictable patellofemoral disorders has been widely studied. A matched case-control study suggests that patellofemoral instability following KA-TKA is related to greater flexion of the femoral component [12]. Some published studies suggest that KA-TKA is more conducive to the restoration of patellofemoral biomechanics than MA-TKA [13, 14], whereas other studies indicate that KA-TKA does not restore the anatomy of the femoral trochlea or increase patellofemoral contact stress [15, 16]. A study based on computer measurements shows that the correlation between natural patellofemoral anatomical parameters and various tibiofemoral alignment parameters is very poor, suggesting that appreciating patellofemoral anatomy will be an important issue in the design of KA femoral prostheses [17].

The above-mentioned studies on patellofemoral disorders related to KA-TKA do not suggest that MA-TKA can perfectly reconstruct the patellofemoral anatomy [11, 12, 15–17]. In contrast, given MA-TKA’s inherent limitations, the distal lateral femoral condyle might be overstuffed, which increases the tension of the lateral retinaculum and is associated with the risk of patellar maltracking [18]. A virtual MA-TKA study suggests that the anterior flange of the femoral component should be lateralized to optimize femoral bone coverage and to eliminate the anteromedial overhang [19]. Another study using virtual resection showed that the geometries of the “Grand piano sign” of anterior femoral resection were different between KA-TKA and MA-TKA, and the ratios of the heights of medial and lateral trochlear resection were approximately 0.7 and 0.6, respectively [20].

To our knowledge, no study has focused on the mismatch between the femoral component and the trochlear resection surface when KA-TKA is performed using conventional prostheses. In clinical practice, we found that undercoverage of the lateral trochlear resection surface in KA-TKA is more common than that in MA-TKA. We hypothesized that this scenario was caused by the design of conventional prostheses, which were developed to comply with the requirements of MA. The reference axes of KA-TKA however are completely different from those of MA-TKA. We hypothesized that the alignment parameters of the lower limbs of different individuals, especially the joint line orientation and the rotation alignment parameters of the distal femur, would have an impact on this undercoverage. Therefore, the aim of the present study was to investigate the correlation between various tibiofemoral alignment parameters and the undercoverage of lateral trochlear resection.

## Methods

### Patients

The Kellgren-Lawrence classification system was used to classify knee OA for each case [21]. Patients with KL grade III or grade IV in the tibiofemoral joint as well as patients with ≤ 5 degrees varus of the proximal tibia (medial proximal tibial angle ≥ 85 degrees), ≤ 5 degrees of valgus deformity were considered for KA-TKA. Exclusion criteria included inflammatory arthritis, previous intra-articular fracture, previous collateral ligament or posterior cruciate ligament rupture, genu recurvatum, and fixed flexion contracture greater than 20 degrees. Given that trochlear dysplasia may have unknown impacts on this study, patients with trochlear dysplasia found preoperatively or intraoperatively were also excluded from this retrospective study.

From May 2018 to August 2020, a total of 55 patients underwent KA-TKA in our institution. After excluding three patients diagnosed with trochlear dysplasia and seven patients with missing intraoperative measurement data of lateral trochlear resection exposure, 45 patients (52 knees) were finally included in this study. Eleven patients were men, and 34 patients were women. The mean age was 69.5 ± 6.7 years (56–85 years), and the mean body mass index was 28.6 ± 4.2 kg/m^2^ (26–34 kg/m^2^). The preoperative radiographic changes of 1 knee were in accordance with Kellgren-Lawrence grade III, and the other 51 knees were in compliance with grade IV. Fifty patients had varus knees, and 2 patients had valgus knees. Among them, 16 patients (16 knees) underwent KA-TKA with the assistance of patient-specific instrumentation (PSI-KA), and 29 patients (36 knees) underwent KA-TKA using conventional instruments with measurement tools (Calipered kinematically aligned instrumentation, Calipered-KA). All bilateral KA-TKAs (seven patients) were performed using the Calipered-KA technique.

### Surgical plans

The design of PSI was based on full-length computed tomography (CT) of the lower extremities, whereas the surgical technique of calipered KA followed the technique recommended by Howell et al. [22]. Regardless of which assistant alignment instrument was used, an articular surface-based bone cut approach was adopted [9]. A Vernier caliper was used to measure the thickness of the resected bone pieces of the distal femoral condyles, posterior condyles, and tibial plateau. The general principle is that the sum of the thickness of the resected bone piece, the compensated thickness of the worn cartilage, and the width of the saw kerf is equal to the thickness of the component.

**Fig. 1:**
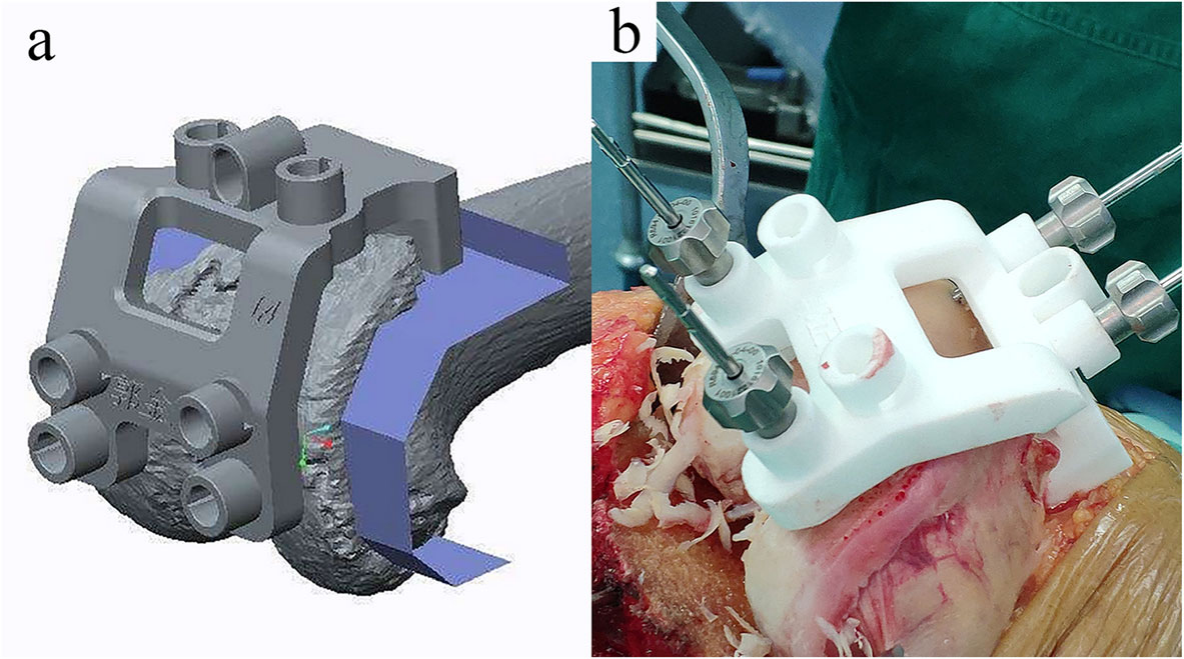
Computer-aided design (**a**) and intraoperative application (**b**) of PSI

To manufacture PSI, CT data (slice thickness, 0.625 mm) were collected and imported into Mimics (version 17, Materialise NV, Belgium) for 3D reconstruction. Then, the solid models were imported into NX 9.0 (Siemens PLM Software, TX, US) for the design of PSI (Fig. 1). Rapid prototyping technology (Formiga P 110, EOS, Krailling, Germany) was used for 3D printing of the PSI. The printing material was medical nylon (PA2200 Polymer powder, EOS, Krailling, Germany), which can be sterilized using autoclaving.

**Fig. 2:**
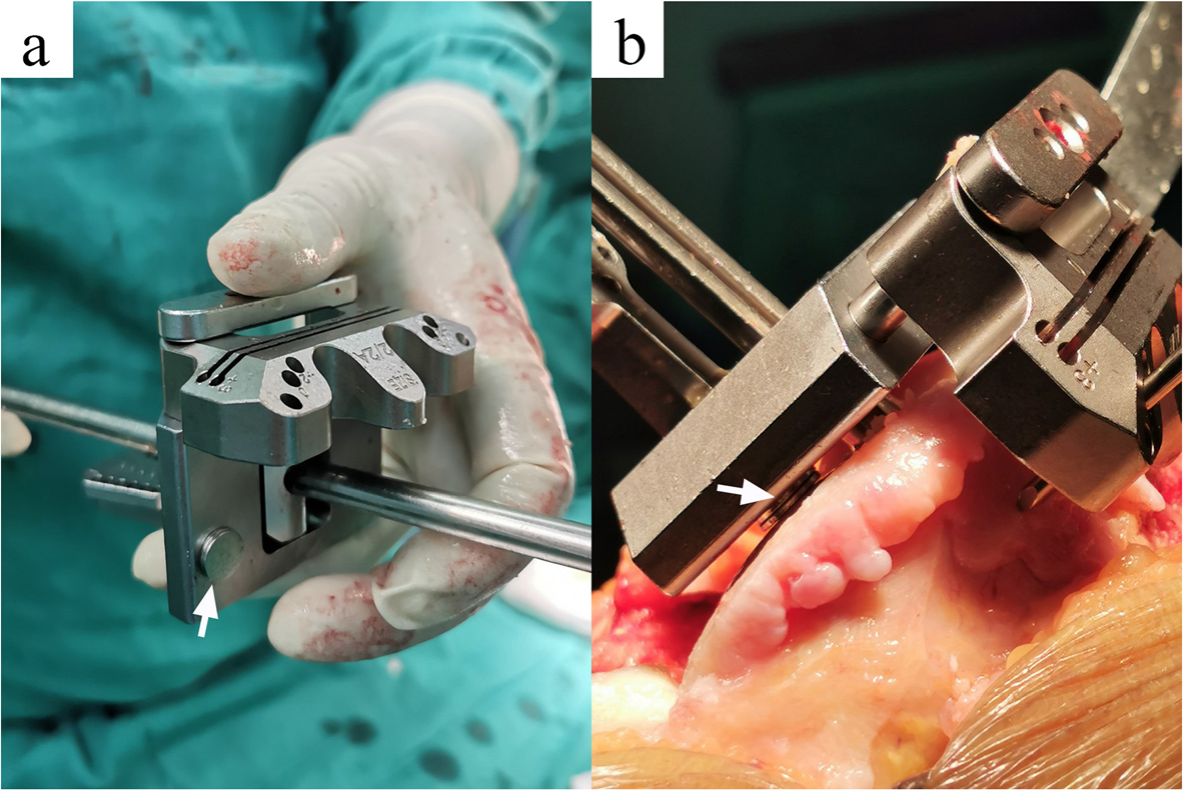
Stacked neodymium magnets (white arrows) are used to compensate for the thickness of worn articular cartilage (2 mm) in calipered KA

For the intraoperative application of PSI, all the residual articular cartilage should be removed using a curette before the PSI is secured to its unique position. In contrast to the management of articular cartilage in PSI-KA, calipered KA only removed the residual cartilage on the severely worn side. If the contralateral articular cartilage was intact, then it was kept in place. Stacked neodymium magnets (1 mm of thickness each) were used to compensate for the cartilage thickness on the severely worn side (Fig. 2). An intramedullary rod was introduced to ensure medial-lateral and flexion-extension orientation of the distal femoral cutting jig. An extramedullary referencing jig was applied on the tibia side.

The distal femoral resection was parallel to the joint line of the distal femur, and the posterior condylar resection was parallel to the posterior condylar axis (PCA). The ‘posterior referencing’ technique was used in femoral bone preparation. The tibial plateau resection was based on the original inclination of the proximal tibial joint line in the coronal plane. The tibial rotation alignment was consistent with the anteroposterior axis of the lateral plateau, and the posterior tilt of resection was consistent with the posterior slope of the medial plateau. A single posterior cruciate retained (CR)-designed prosthesis (Gemini MK II, Link, Hamburger, Germany) was used in the current study. When the undercoverage of the lateral trochlear resection is too obvious, depending on the coverage of distal femoral resection, the femoral component is appropriately lateralized to alleviate the undercoverage of the lateral trochlea. The patella was not resurfaced in any case.

### Parameters measurement

**Fig. 3:**
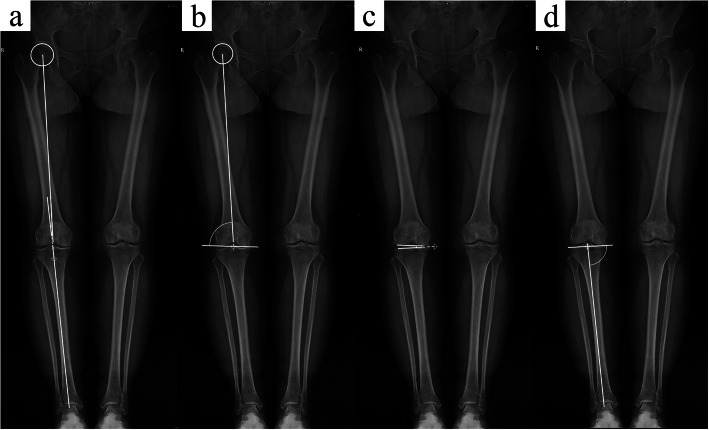
Schematic diagrams for the measurement of tibiofemoral joint anatomical parameters: **a **Hip-knee-ankle angle (HKAA), **b** Mechanical lateral distal femoral angle (mLDFA), **c** Joint line convergence angle (JCLA), and **d** medial proximal tibial angle (MPTA)

**Fig. 4:**
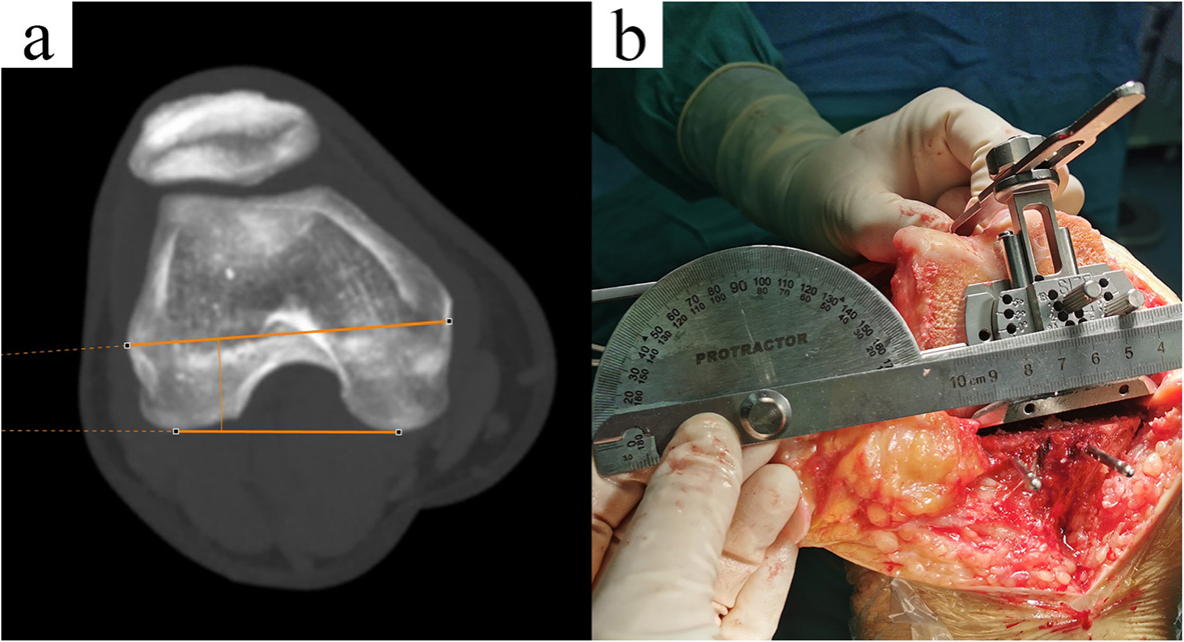
Transepicondylar axis angles (TEAAs) were measured by preoperative superimposed CT (**a**) and a protractor in the operating room (**b**). **a** Determination of the recess of the medial epicondyle, the prominence of the lateral epicondyle, and the highest point of the medial and lateral posterior condyles by the MIP approach of CT. The TEAA value was automatically obtained by the in-house program. **b** Intraoperative measurement required electrocautery to mark the position of the medial and lateral epicondyles on the distal femoral resection surface. The arms of the protractor were parallel to the posterior pedals of instruments and the electrocautery marks

Before KA-TKA, full-length weight-bearing radiographs of the lower limbs were obtained from all patients. The hip-knee-ankle angle (HKAA) was measured from the full-length radiograph. HKAA is defined as the angle between the mechanical axes of the femur and the tibia. The value of varus HKAA is defined as positive, and the value of valgus HKAA is defined as negative. Other alignment parameter measurements followed the methods described by Paley [23]. The mechanical lateral distal femoral angle (mLDFA), medial proximal tibial angle (MPTA), and joint line convergence angle (JLCA) were measured and recorded (Fig. 3). The full-length CT data of all PSI-KA patients were retrieved. The angle between the surgical transepicondylar axis (TEA) and the posterior condylar axis (PCA) was determined using in-house maximum intensity projection (MIP) technology (SOMATOM Sensation, Siemens, Germany), and this angle was defined as the transepicondylar axis angle (TEAA) (Fig. 4a). In addition, TEAA was also measured and recorded using a protractor in all patients included in this study during the operation (Fig. 4b). The above parameters were used as independent variables in this study.

**Fig. 5:**
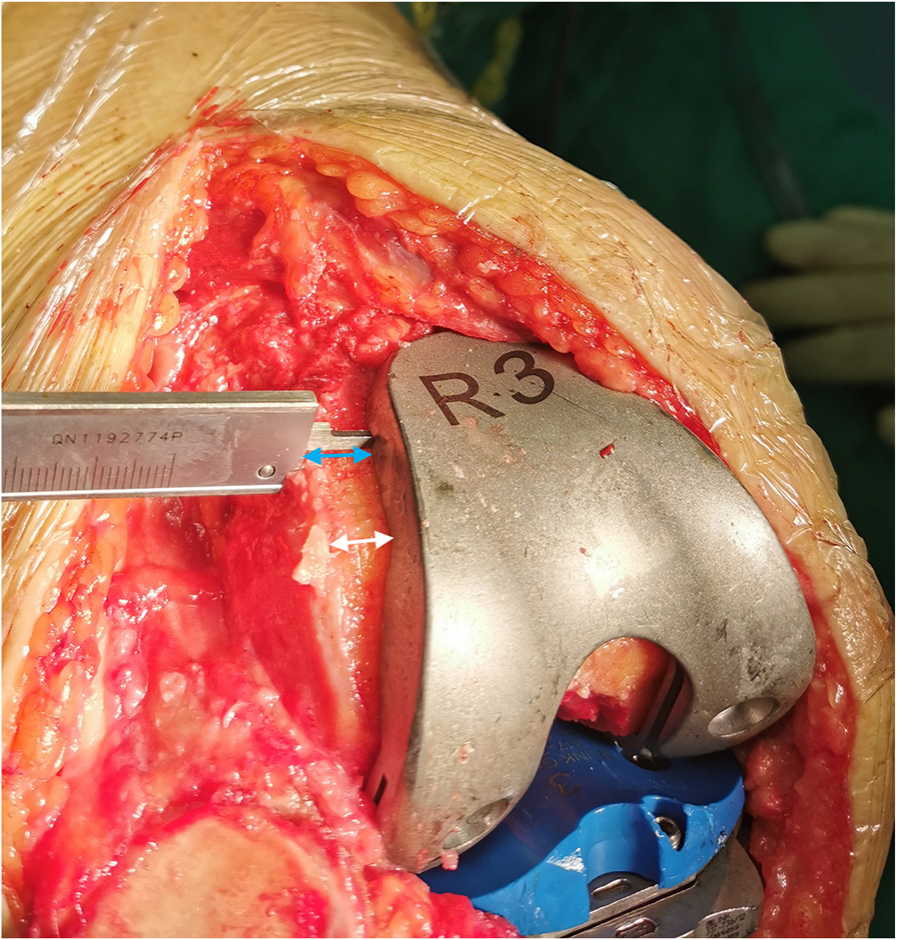
Intraoperative caliper measurement of the exposed bone resection of the lateral trochlea. The white and blue arrows represent the width of the exposed resection at the distal (DISexposure) and middle (MIDexposure) levels of the trochlea, respectively

The distances from the lateral edge of the trochlear resection surface to the femoral component trial were measured at two levels: the corner of the anterior resection and the anterior chamfer resection (distal exposure, DISexposure) and the middle level between this corner and the apex of the anterior flange of the femoral trial (middle exposure, MIDexposure) (Fig. 5). The widths of the exposed trochlear resection at these two levels were used as the dependent variables for subsequent analysis.

### Statistical analysis

All measurement parameters were subjected to the Shapiro-Wilk test. Parameters that conformed to a normal distribution are presented as the mean ± standard deviation, and those that did not conform to a normal distribution are presented as the median (interquartile range). In patients receiving PSI-KA, a reliability test of the two sets of TEAA data from CT measurements and intraoperative measurements was conducted. Reliability was determined by calculating the intraclass correlation coefficient (ICC) with a 95 % confidence interval (CI). An ICC value of > 0.8 indicates very good, 0.6–0.8 good, 0.4–0.6 moderate, and < 0.40 poor[24]. A correlation test was performed between the dependent variables and all independent variables, and linear regression analyses were conducted between variables with significant correlations. All analyses were performed using SPSS (v. 22.0, IBM, Armonk, NY), and *P* < 0.05 was considered statistically significant.

## Results

The mLDFA, MPTA and TEAA conformed to a normal distribution, whereas the other parameters did not. The exposed widths of the distal and middle levels of the lateral trochlear resection surface were 2.0 (2.4) mm (0 ~ 5 mm) and 3.0 (2.9) mm (0 ~ 6 mm), respectively. The other measurements of the respective variables are shown in Table 1. Only 7 knees out of 52 knees had no obvious bone resection exposure of the lateral trochlea, and other knees had various extents of lateral trochlear resection exposure (incidence rate 86.5 %).

**Table 1:** Descriptive statistics of various measurement parameters

	Shapiro-Wilk (Sig.)	Mean ± SD (Range)	Median (Interquartile range) (Range)
HKAA (°)	0.001	NA	6.0 (3.0) (-3.2 ~ 9.6)
mLDFA (°)	0.658	88.2 ± 2.0 (83.7 ~ 93)	NA
JLCA (°)	0.000	NA	4.9 (1.8) (-4.9 ~ 7.4)
MPTA (°)	0.466	87.2 ± 1.3 (85 ~ 90.7)	NA
TEAA (°)	0.093	3.4 ± 1.2 (1 ~ 6)	NA
DISexposure (mm)	0.035	NA	2.0 (2.4) (0 ~ 5)
MIDexposure (mm)	0.023	NA	3.0 (2.9) (0 ~ 6)

*NA *Not applicable

The reliability test (ICC, two-way mixed effects, random, fixed effects) of the two sets of TEAA measurements was performed in 16 patients who received PSI-KA, and the ICC was 0.792 (0.501–0.922), indicating that the two measurement approaches had good consistency. For the data collection of TEAA, the MIP approach of CT exhibits better accuracy than intraoperative measurement [25], so TEAA data of PSI-KA patients are all derived from CT measurements.

**Fig. 6:**
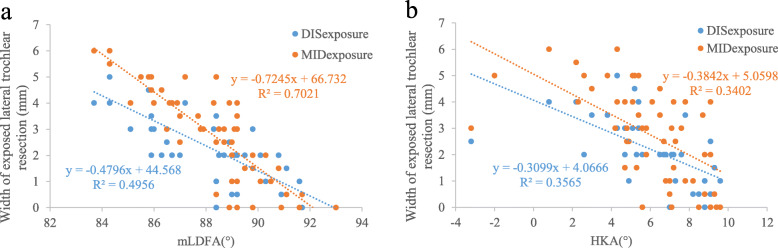
The influence of the distal femoral joint line (mLDFA) (**a**) and the alignment of the lower extremities (HKAA) (**b**) on the undercoverage of lateral trochlear resection, where DISexposure and MIDexposure represent the width of the exposed bone resection at the distal and middle levels of the trochlea, respectively

Spearman correlation analysis results showed that the two levels of lateral trochlear resection exposure were significantly correlated with mLDFA and HKAA but not correlated with other independent variables (Table 2). Further linear regression analyses showed that DISexposure and MIDexposure are more sensitive to the values of mLDFA with regression coefficients of -0.480 (R^2^ = 0.496) and − 0.724 (R^2^ = 0.702), respectively, whereas HKAA has a slight influence on the exposure of the trochlear resection surface. The regression coefficients were − 0.310 (R^2^ = 0.357) and − 0.384 (R^2^ = 0.340), respectively (Fig. 6).

**Table 2:** Nonparametric Spearman’s correlation results among measurement parameters

		HKAA	mLDFA	JLCA	MPTA
MIDexposure	Correlation Coefficient P (2-tailed)	-0.636 < 0.01	-0.834 < 0.01	0.049 0.732	0.129 0.363
DISexposure	Correlation Coefficient P (2-tailed)	-0.646 < 0.01	-0.689 < 0.01	-0.064 0.652	0.242 0.084

## Discussion

The current study confirmed that the extent of exposed lateral trochlear resection during KA-TKA has a correlation with mLDFA. Thus, that the more valgus the joint line of the distal femur is, the larger the lateral trochlear resection surface is exposed. In addition, the extent of exposed lateral trochlear resection is also weakly correlated with the alignment of the lower extremities: the more severe the varus knee is, the smaller the exposed resection surface of the trochlea. The results of this study suggested that the design of a new prosthesis that meets the technical requirements of KA might require lateralization of the anterior flange of the femoral component to a certain extent. Due to significant variation in mLDFA among individuals, such lateralization adjustment should be meticulous; otherwise, in some patients, it could result in lateral overhang of the anterior flange, irritation of the lateral retinaculum and patellar maltracking. The results of this study implied that personalized custom prostheses or modular femoral components may be more promising for the restoration of patellofemoral anatomy in KA-TKA.

Many factors may be related to the undercoverage of the lateral trochlear resection during KA-TKA, such as the joint line orientation of the distal femur (i.e., mLDFA), the rotational alignment of the femoral component (i.e., TEAA), the flexion of the femoral component, the size of the femoral component, the ratio aspect of the distal femur, and other limb alignment parameters (i.e., HKAA, JCLA, MPTA). Among them, the role of mLDFA is the most reasonable. Brar et al. reported that the apex of the anterior flange moves 4 mm medially on average in KA-TKA compared with MA-TKA [26]. Given the valgus joint line of the distal femur in most patients, the femoral component requires more internal rotation on the coronal plane compared with MA-TKA, and then the anterior flange tilts medially and exposes the lateral trochlear resection.

Before this study, we hypothesized that TEAA would also affect the undercoverage of lateral trochlear resection. In terms of femoral component rotation alignment, the reference axes in KA-TKA and MA-TKA are the primary femoral axes (i.e., cylindrical axis) and TEA, respectively. The primary femoral axis is the connecting line between the centers of best-fit spheres of medial and lateral condyles [27], whereas TEA is the most reliable reference axis in the measured resection technique of MA-KTA. The primary femoral axis has been proven to be relatively internally rotated compared with TEA [28]. The rotational alignment of the femoral component significantly affects patellofemoral kinematics; an average 5° of rotation of the femoral component results in a 4° tilt of the patella [29]. However, the results of this study were beyond the preoperative speculation: the extent of exposed trochlear resection is not significantly related to TEAA. This finding indicates that the rotational alignment of the femoral component does not significantly affect the match between the anterior flange and trochlear resection surface. This result may be explained by the fact that the relative internal rotation of the femoral component will only lead to a relative decrease in the amount of lateral trochlear resection rather than significant medialization of the anterior flange.

This study also suggests that HKAA has a minor effect on the undercoverage of the lateral trochlea. HKAA is not only related to the joint line orientation of the distal femur and proximal tibia but is also determined by cartilage loss and soft tissue laxity, which may partially explain why the extent of exposed resection is weakly correlated with the alignment of the lower extremities.

The flexion and the size of the femoral component will also affect the exposure of trochlear resection. Computer simulation analysis shows that the flexion placement of femoral components will cause patellofemoral understuffing, which will increase the width of the trochlear resection [12, 26]. In the calipered KA group of our study, an intramedullary rod was introduced as deep as possible to ensure the flextion-extension orientation so that the position of the femoral component was compliant with the morphology of the distal femur to avoid excessive flexion of the femoral component. Additionally, downsizing of the femoral component results in anterior cortex notching, which subsequently widens trochlear resection. Although Marra et al. demonstrated that downsizing combined with flexing of the femoral component can eliminate anterior cortex notching without bringing additional risks [30], we still double-checked using the ‘angel wing’ before bone cutting.

The mismatch of the femoral component aspect ratio has been widely considered in TKA [31]; this issue is of course no exception in KA-TKA. In this study, if undercoverage was found both in distal femoral resection and lateral trochlear resection, then the femoral component was appropriately lateralized to reduce the extent of lateral trochlear resection undercoverage. From the patellofemoral reconstruction perspective, although lateralization of the femoral component could alleviate the undercoverage of the exposed resection, the adjustment extent of such lateralization is very limited because the coverage of distal femoral resection must be taken into account simultaneously. Concerning irritation of the lateral retinaculum, an oscillating saw was used to trim the prominent trochlear ridges in some patients.

There were several limitations in this study. First, only one type of prosthesis was employed in this study, which does not mean that other commercial prostheses have the same extent of trochlear resection exposure. Further clinical studies involving multiple prostheses or computer simulation studies are necessary to confirm the current results. Second, not all patients had CT data in this study. The majority of TEAA data were acquired from intraoperative measurements, which might impact the accuracy of TEAA. However, the preoperative CT data were retrospectively collected from 16 patients who received PSI-TKA, and the reliability test result of the two sets of TEAAs was “good”. Third, the impact of trochlear resection undercoverage on intraoperative patellofemoral biomechanics, such as patellar tracking (no thumb test, positive or negative) or lateral retinaculum release (yes or no), will provide more valuable information for clinical practice. However, the intraoperative assessment of patellofemoral biomechanics only has binary variables, and the number of patients in the present study is not enough to provide sufficient test power for statistical analysis, including categorical variables. Despite the abovementioned limitations, the results of this study are sufficient to demonstrate that the undercoverage of the trochlear resection surface in KA-TKA is not only related to the design philosophy of the conventional prostheses but also correlated with the degrees of valgus of the distal femur.

## Conclusions

The undercoverage of the trochlear resection surface in KA-TKA is correlated with the degrees of valgus of the distal femoral joint line. The present study suggests that attention should be paid to the impact of tibiofemoral alignment parameters on the match between the femoral component and femoral trochlea resection in the development of KA-specific prostheses.

## Data Availability

The dataset used and/or analyzed in the current study is available from the corresponding author on reasonable request.

## Abbreviations

KA-TKA: Kinematically aligned total knee arthroplasty
MIDexposure: The exposed resection surface at the middle level on the lateral trochlea
DISexposure: The exposed resection surface at the distal level on the lateral trochlea
HKAA: Hip-knee-ankle angle
mLDFA: Mechanical lateral distal femoral angle
JLCA: Joint line convergence angle
MPTA: Medial proximal tibial angle
TEAA: Transepicondylar axis angle
TKA: Total knee arthroplasty
OA: Osteoarthritis
MA: Mechanical alignment
KA: Kinematical alignment
MA-TKA: Mechanically aligned total knee arthroplasty
PSI: Patient-specific instrumentation
Calipered-KA: KA-TKA using conventional instruments and caliper
CT: Computed tomography
PCA: Posterior condyle axis
CR: Cruciate retained
TEA: Transepicondylar axis
MIP: Maximum intensity projection
ICC: Intraclass correlation coefficient
CI: 95% confidence interval
